# Morphology and Luminescence Properties of Transition Metal Doped Zinc Selenide Crystals

**DOI:** 10.1007/s10895-024-04009-9

**Published:** 2024-11-11

**Authors:** Eric Bowman, Leslie Scheurer, Bradley Arnold, Ching Hua Su, Fow-Sen Choa, Brian Cullum, N. B. Singh

**Affiliations:** 1 University of Maryland Baltimore County, 1000 Hilltop Circle, Baltimore, MD 21250 USA; 2 NASA Marshall Space Flight Center, Huntsville, AL 35812 USA

**Keywords:** Zinc selenide, Morphology, Emission, Crystal defects, Physical vapor transport, Optical characterization

## Abstract

Zinc selenide is an excellent matrix material to dope with rare-earth and transition metal to achieve mid-infrared luminescence to develop high power lasers. The luminescence, morphology and refractive index is significantly affected by the doping and defects generated due to size and valency of dopants, concentration, growth process and convection during the growth. The aim of the study is to investigate effect of point and line defects generated due to low doping of iron and chromium on the emission and morphology of the zinc selenide. Luminescence and morphological properties of large iron and chromium doped zinc selenide single crystals were studied to evaluate the effect of extremely low residual impurities and defects associated with the doping process. The emission properties following both short wavelength (i.e., ultraviolet; 350–370 nm) excitation and longer wavelength (i.e., near infrared; 850–870 nm) excitation were characterized. Luminescence emission bands were identified in both doped crystals. In addition to the primary emission bands, satellite peaks and intra-center transitions were also observed. Due to local population defects associated with the residual impurities (ppm to ppb) in the Fe-ZnSe and Cr-ZnSe crystals, peak emission wavelengths were observed to shift. The emission bands were found to decrease in intensity due to recombination of residual impurity co-dopants and complex defects generated during growth and fabrication. Cryogenic temperature analyses revealed a very clean emission band due to freezing of some of the point and line defects. An emission band observed at 980 nm for both crystals at room temperature as well as cryogenic temperatures indicates a vibronic peak in ZnSe. The scanning electron microscopy (SEM) images of the local morphology support the conclusion that small crystallites in doped crystals are also present.

## Background

There is a strong need to replace the rare-earth elements currently used to dope bulk crystals while still achieving the desired emission characteristics for a variety of applications [1–3]. To this end, significant effort is being devoted to investigating the effect of transition metal dopants as potential replacement materials to rare earth doped crystals. Zinc selenide, an excellent bulk crystalline material for doping due to its wide optical transparency range, low absorption coefficient and its ability to readily generate large bulk crystals via physical vapor transport (PVT) methods, is an ideal test matrix for characterizing the potential of alternative transition metal dopants. However, convective forces generated during doped crystal growth are complex [3–5] and can significantly affect the crystal quality due to the roles of both thermal and solutal convection in the growth process. Furthermore, differences in the density, vapor pressure, expansion coefficients and thermal properties between the dopants and the ZnSe can result in diffusive convection known as thermal or double diffusive convection. The resulting point, line and other growth defects associated with these uncontrolled convection phenomena then govern the stacking of atomic layers and in turn the electrical and optical properties of the material [7–21]. Due to the well characterized electronic energy levels of iron (Fe), chromium (Cr), cobalt (Co), nickel (Ni), copper (Cu), vanadium (V), and manganese (Mn), chromium and other transition metal ions doped in PVT grown ZnSe crystals have been demonstrated [6–21] to be potentially attractive optical materials for lasing and other applications. However, since doping creates structural defects and complexity, the transition metal dopant (activator ion) must be employed in as small of a concentration as possible, which is often in contrast to the higher concentrations required to obtain the optimal optical properties. Furthermore, transition metal ions in materials such as chalcogenides exist in several charge states which tend to neutralize in the host materials, resulting in the presence of absorption bands below the fundamental edge and charge transfer between the valence band (or conduction band) and the transition metal ion impurities at these defect sites. This phenomenon, along with the lifetime of the resulting ions, has been described in detail previously [22–34]. The homogeneity and the defects, which have been studied by a variety of methods including SEM, TEM, X-ray diffraction, as well as optical and electrical characterization techniques, have demonstrated that the photoemission effects of the resulting material are altered by the intrinsic defects in the crystals. This report describes the preparation and performance of low very level iron (Fe) and chromium (Cr) doped ZnSe crystals and the analysis of their morphology and spectroscopic studies.

## Experimental Methods

Pure and doped ZnSe crystals were grown in two-zone and three-zone furnaces by PVT methods [4–6]. Detailed optical characteristics of the pure crystals of ZnSe and ZnSe-ZnS have been reported previously [5, 6, 8].

### Morphological Analyses

Morphological analysis of the samples was performed using both optical microscopy and scanning electron microscopy (NOVA NANOSEM 450). Thin SEM samples were evaluated using 3–5 KeV energy electrons, whereas thicker samples we employed up to 20 KeV for data collection.

### Optical Emission Studies

Details of the optical emission measurement employed have been described in detail previously [5, 8, 15, 16]. The emission spectra are strongly related to impurities and defect complexes. Because of this correlation, emission in the range from green to red spectral regions are affected by isolated intrinsic point defects associated with the low concentration dopants, vacancies of zinc, selenium [23–29], complexes formed and centers caused by low residual impurity elements [9, 30–35]. While most previous studies have been performed to understand mid-infrared bands, we used photoluminescence spectra to determine the bandgap and emission in the range of 350–400 nm and 850–870 nm to investigate the effects of the isolated ion-conduction band interactions and photoemission. An inhouse FS920 spectrofluorometer capable of measuring steady state luminescence spectra in the ultraviolet through the near infrared wavelength region was used for this study.

## Results and Discussion

**Fig. 1:**
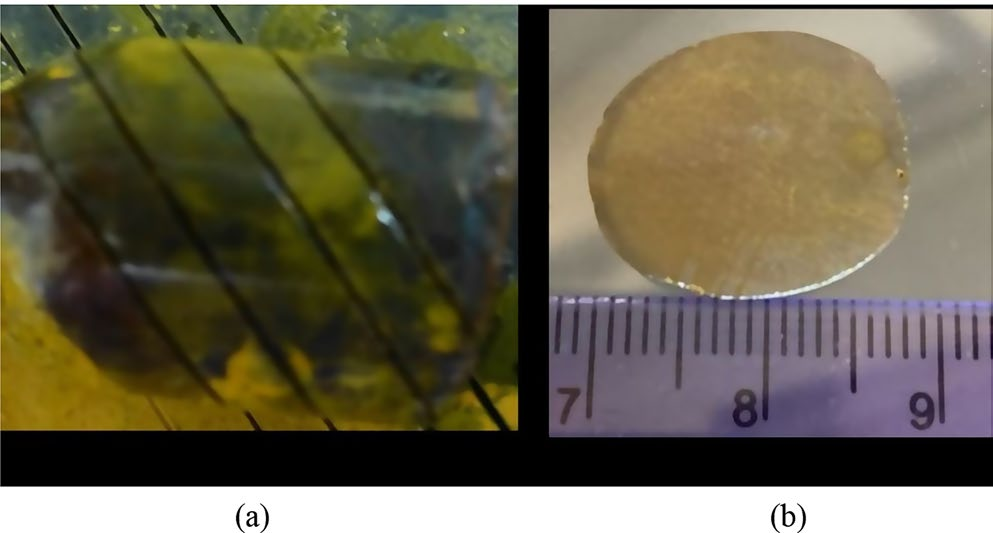
(**a**) Fe-ZnSe crystals grown by physical vapor transport method and (**b**) a slab fabricated in for the study

**Fig. 2:**
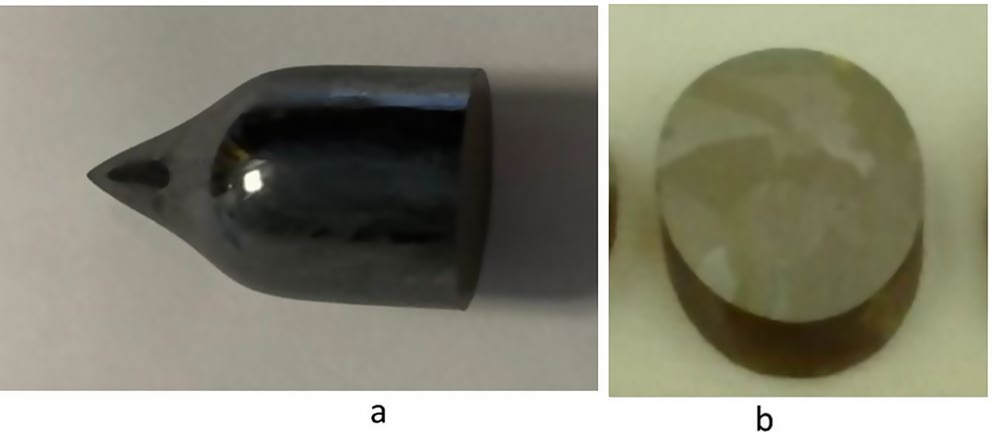
(**a**) Cr-ZnSe crystals grown by physical vapor transport method and (**b**) crystal slab fabricated for the study

Two crystal slabs were fabricated from the large Fe^+ 2^ and Cr^+ 2^ doped crystals grown by the by physical vapor transport (PVT) method in multi-zone furnace in earth- gravity conditions where the thermal and solutal (due to inherent low-level impurities) convections played an important role in controlling the crystal quality. The composition of the ZnSe doped crystals could be given as Zn_1 − x_Fe_x_Se (shown in Fig. 1(a)), where x was 1.8 × 10^− 5^ atomic % and Zn_1 − x_Cr_x_Se (shown in Fig. 1(a)) where x was 4.2 × 10^− 5^ atomic %. The mass of both grown crystals was in the range of 20–22 g. The SIMS analysis showed copper (< 1.7 ppm) and silicon (< 2.1 ppm) impurities in the grown crystals. From these two crystals, cross-sectional slabs approximately 3 mm thick were cut and polished in each side for luminescence and reflection characterization. These Fe- and Cr-doped ZnSe slabs can seen in Fig. 1(b) and Fig. 2(b) respectively. From these images, it can be seen that crystals are free from visible gross defects such as voids, bubbles and precipitates. X-ray diffraction patterns have been discussed in detail and published [3–5] previously.

### Fe-ZnSe crystals

Using these polished crystal slabs, the luminescence properties of the Fe- and Cr-doped ZnSe crystals were characterized. In ZnSe crystals, it is well known that the luminescent bands are associated with transition metal dopants and results from transition metal electrons going into unfilled “d” shell of ZnSe and later returning to the transition metal ion. The 3d^n^ and 4p electron wavefunctions are mixed [17] at asymmetric (non-centro symmetric) sites. This electron transition between crystal field energy levels generally results into IR emission. The ionic radii of dopant Fe^+ 2^ and Fe^+ 3^ are 0.078 nm and 0.063 nm, respectively. The radius of dopant Fe^+ 2^ is larger than that of Zn in ZnSe, which is 0.074 nm, causing point defects and distortion affecting the lattices and overall quality of the crystals. The case of chromium is slightly different since the radius of Cr^+ 2^ is 0.087 nm in coordinate octahedral and 0.094 nm for high spin, and 0.076 nm for Cr^+ 3^ and 0.068 nm for Cr^+ 4^ ions. To achieve high intensity luminescence higher concentration is desirable, however, higher concentration does cause defects and significant deterioration in crystal quality. To characterize the luminescent emission in this study with low dopant concentrations, emission measurements in each samples were obtained in both visible and near-infrared regions following excitations at several UV and near-IR wavelengths. A large number of studies have been performed [17–35] explaining the appearance of transitions in the II-VI materials using UV radiation. Lifetime and other characteristics have been published extensively [19–35], thus we will focus on emission peaks due to different excitation wavelengths.

**Fig. 3:**
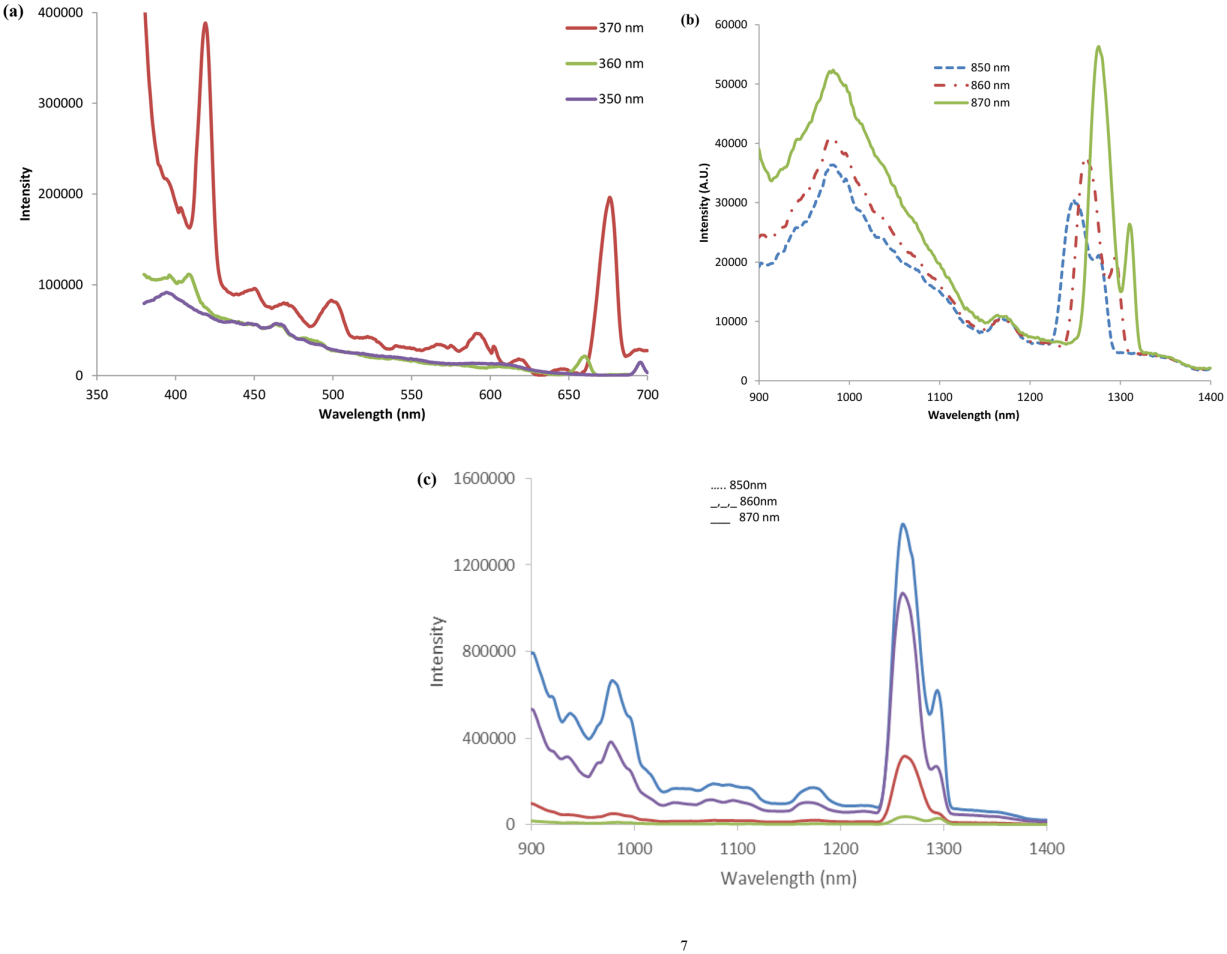
(**a**) Luminescence spectra of Fe-ZnSe using 350, 360 and 370 nm excitation wavelength and a 380 nm filter, (**b**) luminescence spectra of the Fe-ZnSe slab using 850, 860 and 870 nm wavelength excitation and a 645 nm filter, (small intensity peaks in the range of 1250 to 1350 are due to local defect populations), and (**c**) luminescence spectra of a different positions of the same Fe-doped ZnSe slab 850, 860 and 870 nm wavelength excitation and a 645 nm filter wavelength. It shows peaks at similar positions to that shown in Fig. 3(**b**)

Figure 3(a-b) show the results for the Fe-doped ZnSe slab. As can be seen from Fig. 3a (360 nm excited spectrum), the emission for this Fe doped ZnSe crystal was 428 nm (2.89 eV) indicating that the bandgap was higher than pure ZnSe (2.54 eV). In addition, the 420–430 nm emission band observed following excitation, has been described as the emission associated with an electronic transition from an exciton to native defects present in the crystal [35]. However, unlike previous studies of highly doped ZnSe crystals [12, 13, 24–28], the low dopant levels associated with this crystal result in a narrow band that is easily resolved from the other photoluminescent emission sites of different energies, thereby providing distinct identification of these transitions without the need for spectral deconvolution. Confirmation of the presence of isolated energy selected centers being associated with these luminescent transitions is revealed from the energy shifting of the 420–430 nm and 675–690 nm emission band after excitation with 360 and 370 nm light. Since emissions all occur between energy selected states, they shift according to the excitation energy. In addition to probing the photoluminescent emission in the visible region of the electromagnetic spectrum, the near-infrared photoluminescence was also measured (Fig. 3b) following excitation at 850, 860 and 870 nm. In these studies, a 645 nm long pass filter was used to filter the excitation light prior to being absorbed by the sample to prevent any second order excitation of the sample via UV/visible light. Since the band observed around 990 nm not reported earlier, we had performed measurements for a doped crystal at cryogenic temperature were performed to ensure its presence.

The band at 990 nm has been assigned to a d – d transition in Fe^+ 2^. The shifting bands between 1200 and 1300 nm are again characteristics of intra-center transitions and complex defects composed of zinc vacancy ions with the shifts in the peak maxima attributed to the excitation wavelengths employed and the different defect sites formed in the crystal. The weak emission bands are usually attributed to the emission of bound excitons, while the other transitions are related to the emission of Fe^+ 2^ and impurity-defect complexes [28] in MBE grown samples. Low intensity bands have been observed [5, 12, 28, 35] in pure and doped ZnSe at room temperature due to extremely low impurities and high temperature growth in quartz containers by physical vapor transport method, and some point defects due to convective transports.

As the size of crystals increases from nano to bulk size [36, 37, 38], it is very important to understand how the segregation both radially and longitudinally, affects optical behavior. To investigate effect of segregation and surface variations in large single crystals, we characterized the near-infrared photoluminescence properties at several locations across the polished slab as well as obtained scanning electron microscope (SEM) images of the crystals. In the emission studies, 870 nm excitation was employed along with the 645 nm long-pass filter to reject any potential second order excitation. As before, broadband emission between 925 and 1000 nm as well as the two primary bands at 1280 nm and 1310 nm are present for each of the four different locations probed, with only the absolute intensity changing from location-to-location, suggesting that the defect and Fe ion replacement sites are at very low concentrations and non-homogenously dispersed across the crystal.

**Fig. 4:**
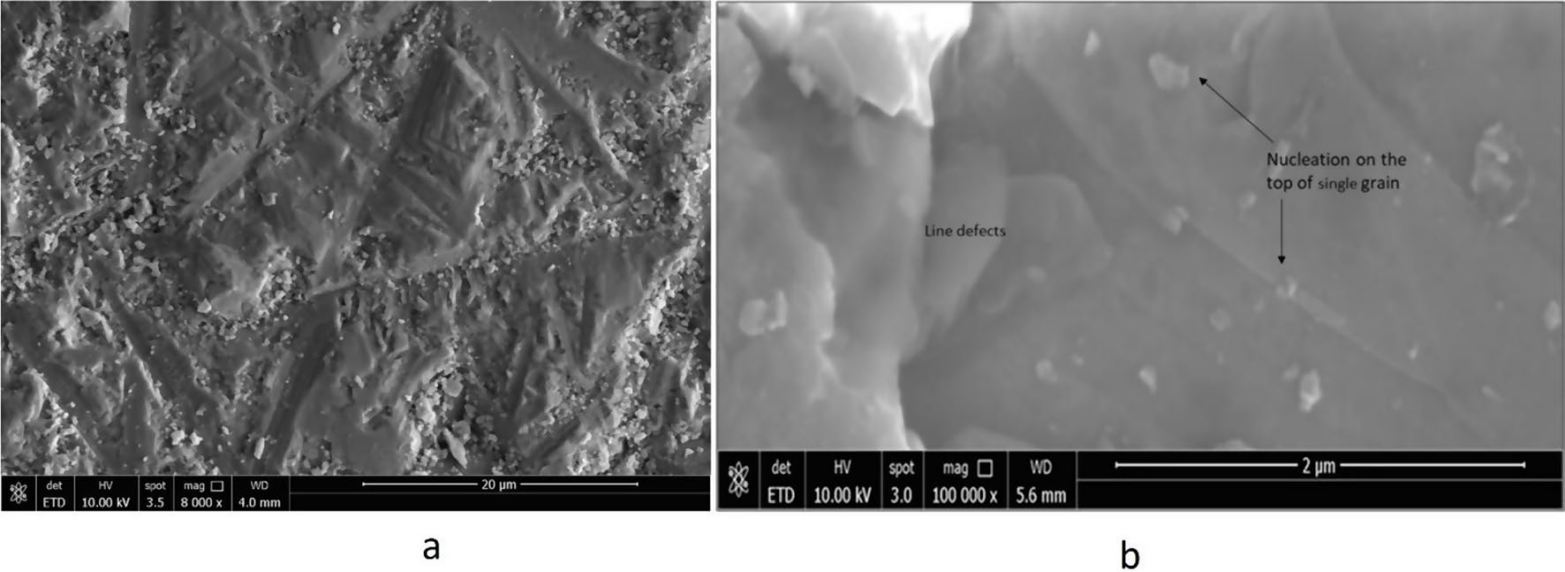
(**a**) Morphology of Fe-ZnSe crystal using 10 KV and (**b**) morphology of crystal shows local concentration of line defects between the grains

The uniformity was evaluated by determining morphology using SEM images (Fig. 4a and b) The images shown are taken at 5 kV at low and high magnification, respectively. Figure 4a shown at lower magnification line and point defects and formation of faceted shapes, in the 40 μm x 60 μm section imaged in Fig. 4a, multiple line defects in the single crystal can be seen and when magnified further (Fig. 4b), it is possible to see the multiple nucleation sites on a single grain of the crystal surface. Large grains of several micrometers are stacked up and show line defects. Small nuclei of extremely small size are visible on large grains. These nuclei were faceted and filled the gap by a layered growth mechanism.

### Cr-ZnSe Crystals

**Fig. 5:**
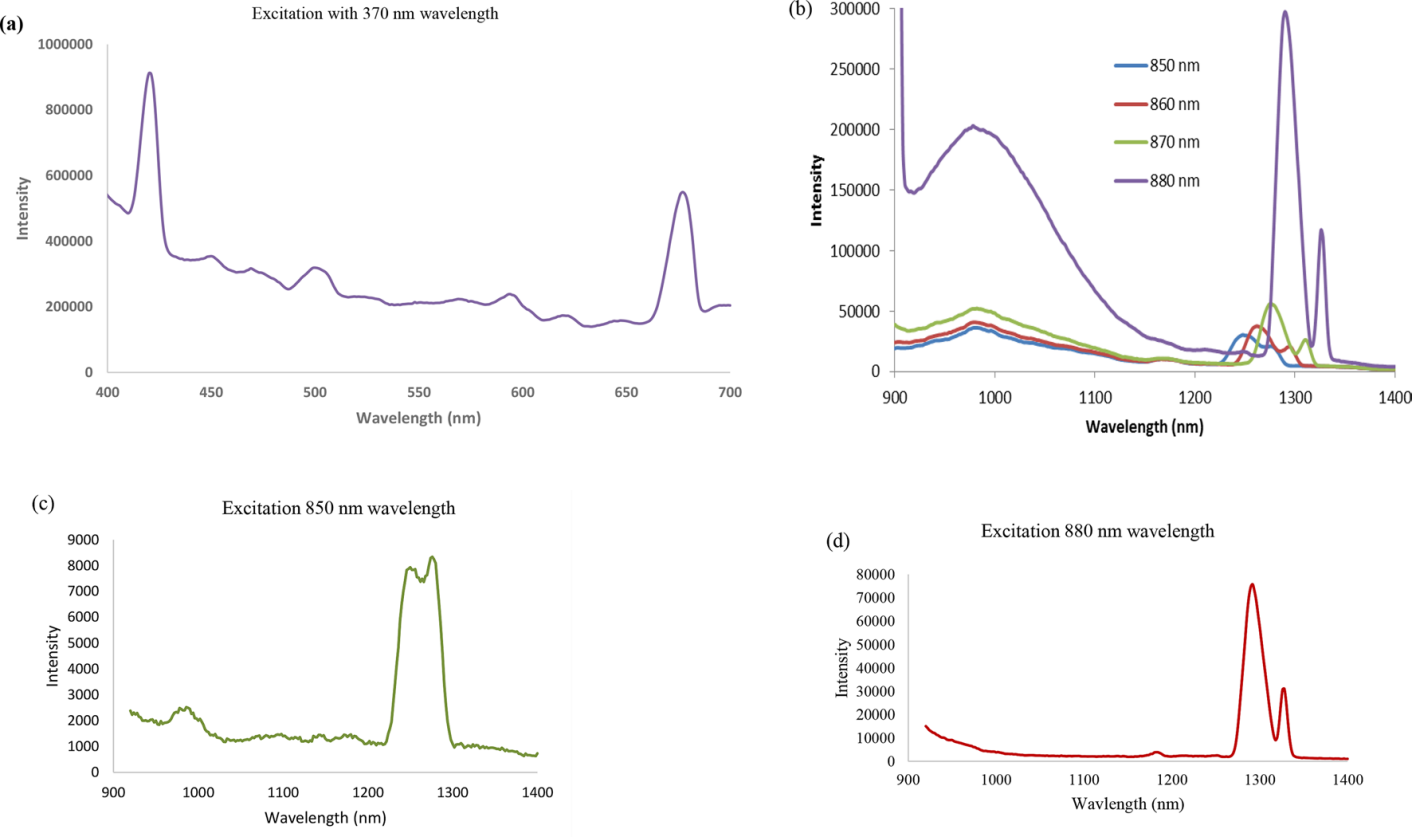
(**a**) Emission spectra of the Cr-doped ZnSe excited at 370 nm with 380 nm filter, (**b**) Luminescence spectra of different positions in the Cr-ZnSe using 850, 860, 870 and 880 nm excitation wavelength and 380 nm emission filter, (**c**) luminescence for Cr-ZnSe crystal with 850 nm excitation wavelength and a 645 nm filter showing peak splitting between 1225 and 1295 nm, and (**d**) luminescence spectra at different position than position of emission shown in Fig. 5(**c**) in the Cr-ZnSe excited using 880 nm and a 645 nm wavelength filter

Similar to the case of Fe-ZnSe, the Cr-ZnSe sample was also characterized for morphology and photoluminescent properties to understand the effect that isolated ion-induced defect sites have on its optical characteristics as compared to doped crystals with large dopant concentration. The photoluminescence emission characteristics of the Cr-ZnSe crystal slab was analyzed in both the ultraviolet/visible spectral region as well as the near-infrared, with specific excitation wavelengths employed to determine the energy isolated transitions and the non-ion related emission transitions. The Cr-ZnSe crystal slab revealed excitation wavelength shifted emission bands and non-shifted bands with varying magnitudes depending on the excitation wavelength employed. The emission bands shown in Fig. 5(a) for Cr-ZnSe are similar to that of Fe-ZnSe shown in Fig. 3, the most prominent bands and greatest photoluminescence intensity was observed following 370 nm excitation.

From this 370 nm excited spectrum shown in Fig. 5(a), it can be seen that the Cr-ZnSe crystal also exhibits 411 nm cutoff (3.01 eV), again indicating a higher bandgap than the pure crystal. As in the Fe-doped sample, the observed transitions are due to native crystal defect sites. Like the previous Fe doped ZnSe measurements, the low concentration of Cr doped into the ZnSe crystal in this study (as compared to previous work [12–13, 24 − 18] with high dopant concentrations), the luminescent emission bands in this work are significantly narrower, making each band easy resolved from the others without the need for computational deconvolution of the contributing bands.

In addition to probing the emission in the visible region of the electromagnetic spectrum, the near-infrared photoluminescence was also measured. When the Cr-ZnSe slab is excited with 850 nm light and the near-infrared photoluminescence spectrum is measured (Fig. 5b), a similar set of emissions are observed between 1200 and 1300 nm. The broadband emission for Fe-ZnSe between 925 and 1125 nm is present in Cr-ZnSe suggesting that Cr ions resulting in a radiative relaxation of the transition. Some investigators have observed double peaks (Fig. 5c and d) in copper zinc selenide. They attributed this to Se^− 2^ (mono selenide) with double separation of 0.11 eV for the Cu_2_Se with Cu/Se molar ratio. This is very important since in both cases (Fe and Cr-doped ZnSe) we observed similar emission peaks approximately at 1.26 and 1.29 μm emission energies. The slight difference in positions may be attributed to the point or line defects and combinations of all type of defects in crystals caused by impurities.

The emissions shown in Fig. 5(c) and 5(d) for the crystal taken at two different positions of the crystal using 850 nm and 880 nm excitation wavelengths showed peak splitting. A slight difference in the position of the emission is attributed for local defect concentrations, but further study is required explore it.

**Fig. 6:**
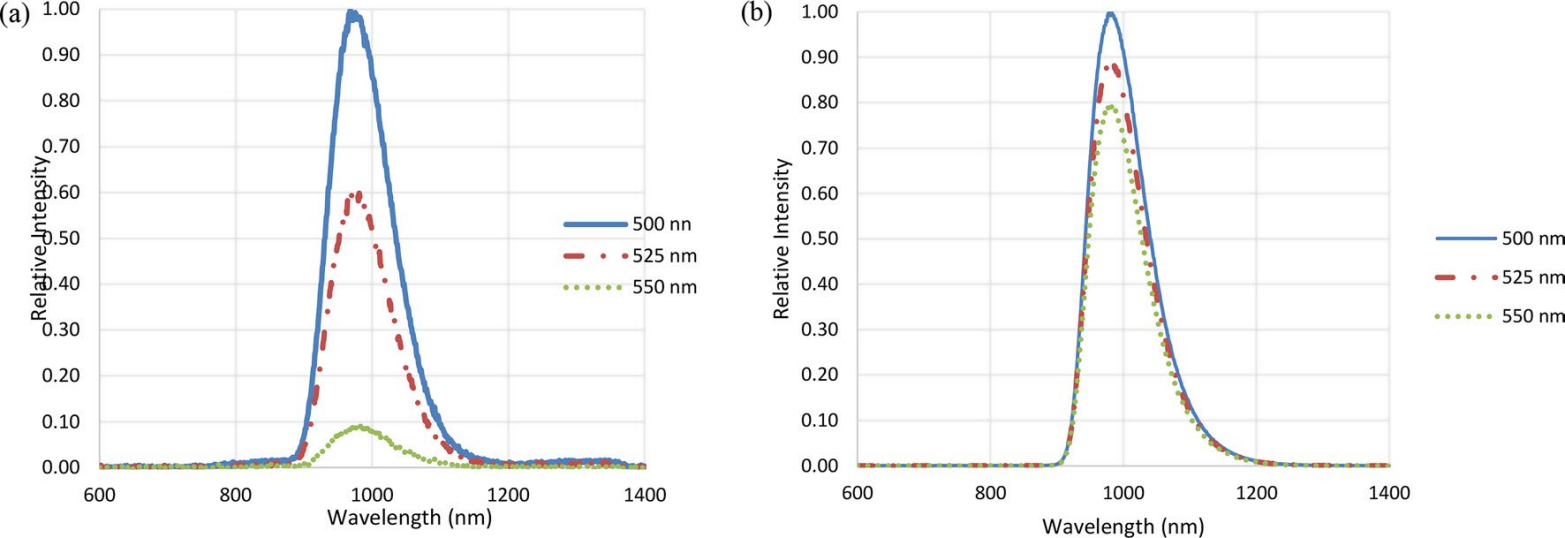
(**a**) Luminescence spectra for Fe-ZnSe crystal and (**b**) Cr-ZnSe crystal at cryogenic temperature using three different excitation sources of 500, 525 and 550 nm wavelength. We used these wavelengths to evaluate if there were any other emissions in range of 600 to 900 nm range

We observed very high intensity emission in both Fe-ZnSe and Cr-ZnSe crystals near 980 nm attributed to the residual impurities and unintentional residual impurities related co-dopants. This peak requires further attention since it is not clear what causes this peak in both crystals. Using coolants, it is possible to freeze some defects and trapped impurities, so we studied this region at cryogenic temperature. The results of emission study at cryogenic temperature are shown in Fig. 6(a) and (b). As we have indicated earlier that SIMS and EDX data both showed the presence of some residual Si (less than 1 ppm) impurities in Fe-ZnSe and Cr-ZnSe samples, possibly coming from the quartz containers during growth at high temperatures. Behnaz et al. [18] explained that this emission may be due to very small concentration of co-dopants which may cause single excited states with energy difference to the ground state.

**Fig. 7:**
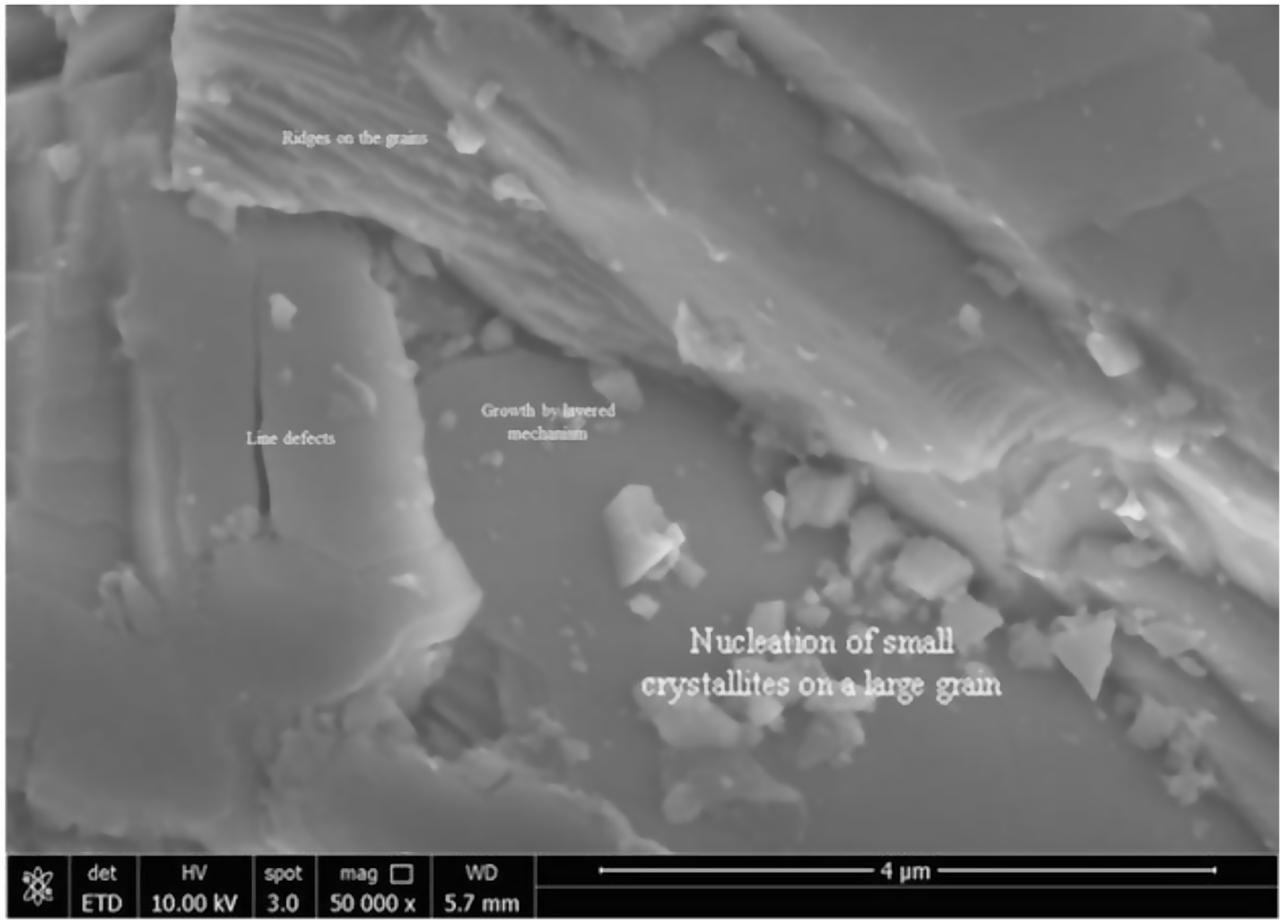
Morphology showed nucleation of faceted crystals on the top of large grains causing difference in defect variation in the crystal and showing nucleation of small crystallites and line defects

To further elucidate the defects, detailed SEM studies were performed for Cr-ZnSe crystal. Figure 7 shows the morphology and nucleation sites of faceted crystals on the top of large grains, causing a difference in defect distribution in the crystal. The nucleus sizes varied in the range of 50 to 100 nm. This was observed after cooling, and we expect that during growth the size may be much smaller. Defect population variation and the presence of smaller nuclei on the top of large grains and line defects can generate weak emission peaks. The difference in the intensity is attributed to the point and line defects, grains, broken facets and other factors governing crystal quality.

The absorption in both doped crystals, values observed were similar to that of with published data. The absorption plots with bandgaps estimates at 2.76 eV (450 nm) for the Fe-ZnSe crystals and at 2.88 V (430 nm) for the Cr-ZnSe crystals were slightly higher than for known bandgap of 2.70 eV for the pure ZnSe. The difference for these very low doped crystals may be attributed to crystal quality of pure and doped zinc crystals.

## Summary

Iron and chromium doped zinc selenide bulk crystals were studied to evaluate the effect of defects generated due to doping and to observe emission peaks due to defects and recombination’s of defects. Emission studies were done using 350, 360 and 370 nm excitation in and 850, 860 and 870 nm excitation wavelengths. Emission studies showed very good crystal quality of the large PVT grown crystal. In addition to main peaks, satellite peaks and vibronic characteristic of intra-center transfer transitions were observed indicating the presence of emitting complex defects composed of zinc vacancies in doped crystals. An emission peak around 980 nm was studied for the first time at cryogenic temperature and was attributed to an extremely low concentration of impurities coming from the growth ampoule or from solvents during cleaning of the growth ampoules.

## Data Availability

No datasets were generated or analysed during the current study.
